# The Janus face of *Bifidobacterium* in the development of atopic eczema: A role for compositional maturation

**DOI:** 10.1111/pai.70041

**Published:** 2025-02-11

**Authors:** Martin Depner, Diana Hazard Taft, Stefanie Peschel, Caroline Roduit, Anne M. Karvonen, Cindy Barnig, Amandine Divaret‐Chauveau, Josef Riedler, Juha Pekkanen, Elisabeth Schmausser‐Hechfellner, Giulia Pagani, Roger Lauener, Marjut Roponen, Harald Renz, Petra Ina Pfefferle, Bianca Schaub, Erika von Mutius, Pirkka V. Kirjavainen, Markus J. Ege, the PASTURE study group, M. Täubel, ML. Dalphin, L. Laurent, C. Beerweiler, A. Böck, F. Foppiano, AJ. Hose, S. Illi, S. Pechlivanis, J. Theodorou, R. Frei

**Affiliations:** ^1^ Institute of Asthma and Allergy Prevention, Helmholtz Zentrum Munich, German Research Center for Environmental Health Neuherberg Germany; ^2^ Food Science and Technology University of California Davis California USA; ^3^ Christine Kühne Center for Allergy Research and Education (CK‐CARE) Davos Switzerland; ^4^ Children's Hospital of Eastern Switzerland St. Gallen Switzerland; ^5^ Division of Respiratory Medicine and Allergology, Department of Paediatrics, Inselspital University of Bern Bern Switzerland; ^6^ Department of Health Security National Institute for Health and Welfare Kuopio Finland; ^7^ Department of Respiratory Disease University Hospital of Besançon Besançon France; ^8^ INSERM, EFS BFC, UMR1098, Interactions Hôte‐Greffon‐Tumeur/Ingénierie Cellulaire et Génique University of Franche‐Comté Besançon France; ^9^ Pediatric Allergy Department Children's Hospital, University Hospital of Nancy Vandoeuvre les Nancy France; ^10^ EA 3450 DevAH, Faculty of Medecine University of Lorraine Vandoeuvre les Nancy France; ^11^ UMR/CNRS 6249 Chrono‐Environnement University of Bourgogne Franche‐Comté Besançon France; ^12^ Children's Hospital Schwarzach Schwarzach Austria; ^13^ Department of Public Health University of Helsinki Helsinki Finland; ^14^ University of Zurich Zurich Switzerland; ^15^ School of Medicine University of St Gallen St Gallen Switzerland; ^16^ Department of Environmental and Biological Sciences University of Eastern Finland Kuopio Finland; ^17^ Institute for Medicine Laboratory, Pathobiochemistry and Molecular Diagnostics, Philipps‐University Marburg Marburg Germany; ^18^ German Center for Lung Research (DZL) Sites Marburg and Munich Germany; ^19^ Comprehensive Biobank Marburg (CBBMR), Philipps‐University Marburg Marburg Germany; ^20^ Dr. von Hauner Children's Hospital Ludwig Maximilians University Munich Munich Germany; ^21^ Institute of Public Health and Clinical Nutrition, University of Eastern Finland Kuopio Finland

**Keywords:** atopic eczema, *Bifidobacterium*, compositional maturation, late‐onset rash, persistent rash, subgenus composition, transient rash, window of opportunity

## Abstract

**Background:**

Atopic eczema often develops in the first year of life, when the composition of the gut microbiota is most plastic as illustrated by the decrease in bifidobacteria after weaning. This may provide the opportunity for microbial stimuli and their environmental determinants to alter the disease course.

**Objectives:**

To determine the role of the genus *Bifidobacterium* for atopic eczema in early childhood.

**Methods:**

We analysed the bacterial composition in fecal samples of 618 children of the PASTURE (“Protection against Allergy—Study in Rural Environments”) birth cohort using 16S rRNA amplicon sequencing of fecal samples collected at 2 and 12 months of age. Atopic eczema was defined as a parent‐reported doctor's diagnosis until 2 years, and patterns of rash symptoms were classified by latent class analysis. We applied mediation models to assess direct and microbiota‐mediated effects of environmental determinants on atopic eczema.

**Results:**

The *Bifidobacterium* composition observed at 2 months was inversely related to atopic eczema (OR = 0.68 [0.53–0.87], *p* = .002) and persistent rash. This association was not seen at 12 months, when the composition of *Bifidobacterium* amplicon sequence variants (ASVs) was altered. The effect of beneficial ASVs at 2 months (OR = 0.72 [0.57–0.91]) was lost at 12 months (OR = 0.97 [0.76–1.24]), when distinct bifidobacteria tended to be positively related to late‐onset rash.

**Conclusions:**

The subgenus composition of *Bifidobacterium* undergoes substantial changes in the first year of life. The protective effect of *Bifidobacterium* depends on the ASV composition at the respective age of the infant, highlighting the importance of timing in prevention strategies targeting infant‐microbe interactions.

**Key message**

The composition of amplicon sequencing variants (ASV) within the genus *Bifidobacterium* undergoes substantial changes in the first year of life. The protective effect of *Bifidobacterium* depends on the ASV composition at the respective age of the infant and an early window of opportunity, which is closer to 2 rather than 12 months of life. This might explain why several intervention studies for atopic eczema failed: Either they might have used the wrong *Bifidobacterium* strains or beneficial *Bifidobacterium* strains might have been applied after the window of opportunity had closed.

## 1 INTRODUCTION

Perturbation of the gut microbiome is associated with several chronic diseases such as atopic eczema (AE).^1^, ^2^ Disruption of the microbial homeostasis in the gut involves immunologic, metabolomic and neuroendocrine pathways associated with the gut‐skin axis.^3^

AE often begins in the first year of life and follows different trajectories,^4^, ^5^, ^6^, ^7^, ^8^, ^9^ opening various windows of opportunity for microbial stimuli to alter the disease course and to provide a potential to determine life‐long health.^9^, ^10^, ^11^ One consistently demonstrated illustration of this effect is the long‐lasting protection against asthma by a timely maturation of the gut microbiome in the first year of life,^12^, ^13^ which was recently extended to AE and other allergic diseases.^14^

The formation of gut microbiota begins at birth, and it has the greatest compositional plasticity in the first year of life when it transits from a *Bifidobacterium*‐dominated composition, seen in exclusively breastfed infants, towards increased complexity during weaning.^15^ Thereafter, microbes continuously diversify in species abundance until age 2–3 years, whereupon the microbiota resemble an adult‐like pattern.^16^

Though the predominant presence of *Bifidobacterium* at a critical period of immune maturation is well known, its involvement in the bacterial maturation process and relation to AE remains controversial.^17^, ^18^

Here, we sought to evaluate how compositional maturation within *Bifidobacterium* influences the development of AE in early childhood. The Protection against Allergy—Study in Rural Environments (PASTURE) cohort offered the opportunity to study the interplay of environmental determinants, the composition of the gut microbiome, and the development of AE in a large multi‐center birth cohort.

## 2 METHODS

### 2.1 Study population

The PASTURE birth cohort enrolled children from rural areas of Austria, Finland, France, Germany, and Switzerland, of whom 50% were born to mothers living on a family‐run livestock farm.^19^ Briefly, women were invited to participate during their third trimester of pregnancy. Their children were recruited at birth and followed up for at least 6 years.^20^ Additional detailed information on the children's health, nutrition, and farm‐related exposures was gathered by using weekly diaries and monthly questionnaires covering the 9th to 52nd weeks of life (Appendix S1).^20^, ^21^, ^22^

All aspects of the study were approved by the local institutional review boards in each country (Austria: Ethikkommission für das Land Salzburg; Finland: The Research Ethics Committee, Hospital District of Northern Savo; Germany: Ethik‐Kommission der Bayerischen Landesärztekammer; Switzerland: Kantonale Ethik‐Kommission St. Gallen; France: Comité Consultatif pour la Protection des Personnes en Recherche Biomédicale (CCPPRB) Commission Informatique et Libertés (CNIL)). Written informed consent was obtained from the parents or guardians.

### 2.2 Health outcomes

Atopic eczema (AE) was defined as a parent's report of a doctor's diagnosis until 2 years, if not further specified. A parent's report of a doctor's diagnosis of AE ever at 6 years of age was additionally used in sensitivity analyses. Itchy rash was defined as parent‐reported itchy rash with scratching or rubbing the skin since the last follow‐up at 12, 18, 24, 36, 48, 60, and 72 months, respectively.

### 2.3 Microbiome analyses

Fecal samples were collected from the child's diaper at 2 and 12 months. DNA was extracted from the fecal samples by the bead‐beating method (Appendix S1). Amplification and sequencing of the V4 region of the 16S rRNA gene were performed as described previously using primers F515 and R806.^23^, ^24^, ^25^, ^26^ Sequencing was performed on an Illumina MiSeq instrument in multiple runs. The resulting reads were denoised using DADA2 as implemented in QIIME2 (https://docs.qiime2.org/2024.10).^27^, ^28^ Taxonomy was assigned using a Naive Bayes classifier trained on the Silva 138 99% database, which was largely compatible with a previous classification using Greengenes (Figure S1).^12^

### 2.4 Statistical analysis

Statistical analysis was performed with R 4.3.1,^29^ particularly with the phyloseq package.^30^ Latent class analysis (LCA) on rash from 1 to 6 years was performed using the R package poLCA.^31^

Similar to the eigengene analysis,^32^ we used the first axes of genus‐specific principal component analyses (PCA) on ASVs at months 2 and 12, respectively. In a joint PCA, ASVs of month 2 and month 12 of the genus *Bifidobacterium* were entered in the same PCA. Regression models were used to determine associations between microbial variables and the outcomes or the environmental determinants. To compare indirect and direct effects, mediation models were calculated in Mplus.^33^ Network analyses were carried out using the R package NetCoMi (v1.0.3).^34^

## 3 RESULTS

### 3.1 Study population and health outcomes

Of the 930 children of the Austrian, Finnish, German, and Swiss PASTURE subpopulations, 618 children (66%) had full information for 16S rDNA from fecal samples collected at 2 and at 12 months (Figure S2). In the 565 children (95%) with data on AE (Table S1), the prevalence of a doctor's diagnosis of AE until 2 years was 15%. AE was already diagnosed in 0.5% and 8.5% of children at 2 and 12 months, respectively. When asked at age 6 years, parents recalled an AE diagnosis in 10% of the participants. For a more symptom‐based approach to AE, we performed a latent class analysis (LCA) for itchy rash and detected a persistent (6% of all children) and a transient (10%) class, whose course was indistinguishable during the first 2 years but separated clearly thereafter (Figure S3). At about the same time point, a late‐onset (12%) class emerged.

### 3.2 The development of *Bifidobacterium* within the first year

**FIGURE 1:**
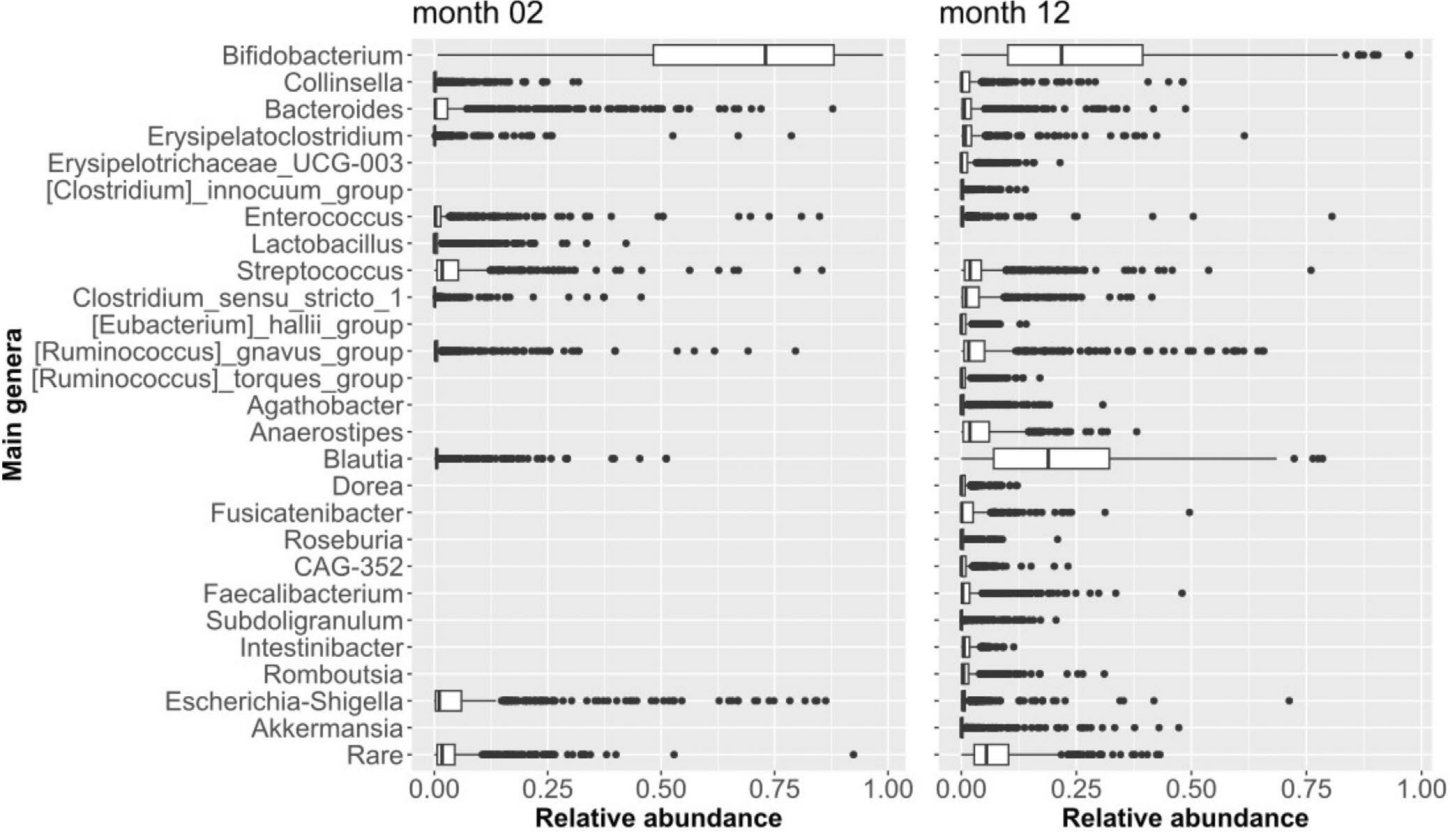
Composition of the gut microbiome at 2 and 12 months. Shown are the relative abundance values of the common (mean relative abundance ≥0.5%) genera at 2 or 12 months. “Rare” summarizes all taxa with a mean relative abundance below 0.5% at 2 or 12 months, respectively. *N* = 618. Lower and upper hinges of the boxes denote the first and third quartiles, respectively; the bold central line represents the median; the whiskers extend to the most extreme data point within a distance of 1.5 times the interquartile range from the hinges; extreme values lie beyond the whiskers and are marked by circles.

Figure 1 illustrates the relative abundance of all common genera at both time points. As expected, the gut microbiota at 2 months were clearly dominated by the genus *Bifidobacterium*, whereas at 12 months the composition was more diverse, although *Bifidobacterium* still had the highest mean relative abundance (27%). Because of the predominance of *Bifidobacterium*, we focused on this genus and calculated for each time point a PCA based on all ASVs within the genus *Bifidobacterium* and used the primary axes (PA‐2, PA‐12) of the PCAs as representants of the *Bifidobacterium* composition at the respective time points.

**FIGURE 2:**
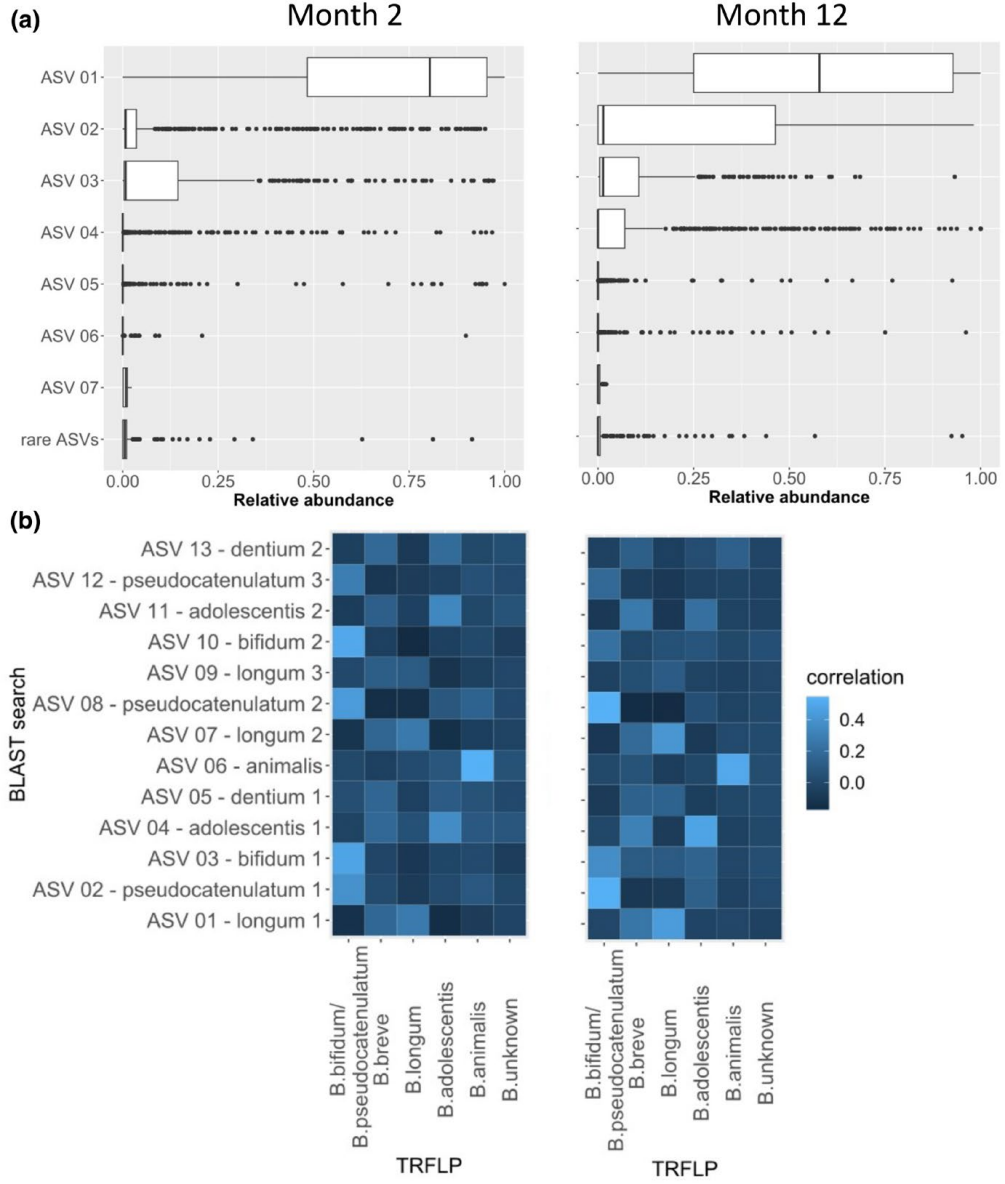
*Bifidobacterium* ASVs at 2 and 12 months. (A) Boxplot of the relative abundance of the most abundant (ASV01 to ASV07) and low abundant ASVs (summation variable, mean relative abundance <0.5%) within the genus *Bifidobacterium* only. Lower and upper hinges of the boxes denote the first and third quartiles, respectively; the bold central line represents the median; the whiskers extend to the most extreme data point within a distance of 1.5 times the interquartile range from the hinges; extreme values lie beyond the whiskers and are marked by circles. For description of ASVs see Table S2, *N* = 618. (B) Correlation of ASVs with TRFLP with respect to relative abundance. ASVs are named according Table S2. *N* = 618.

Different ASVs correlated with PA‐2 and PA‐12 (Figure S4). PA‐2 correlated most strongly with ASV‐03, which was among the most common ASVs at 2 months (Figure 2A) and was assigned to *B. bifidum* by an exploratory BLAST search (Table S2) supported by Terminal Restriction Fragment Length Polymorphism (TRFLP, Figure 2B).

PA‐12 correlated most strongly with ASV‐02 and ASV‐04, which were compatible with *B. pseudocatenulatum* and *B. adolescentis*, respectively. The correlations between the main ASVs of Bifidobacterium were also different between the two time points as illustrated by differentially strong edges of the corresponding networks (Figure S5).

### 3.3 The effect of Bifidobacteria on atopic eczema within distinct time windows

**FIGURE 3:**
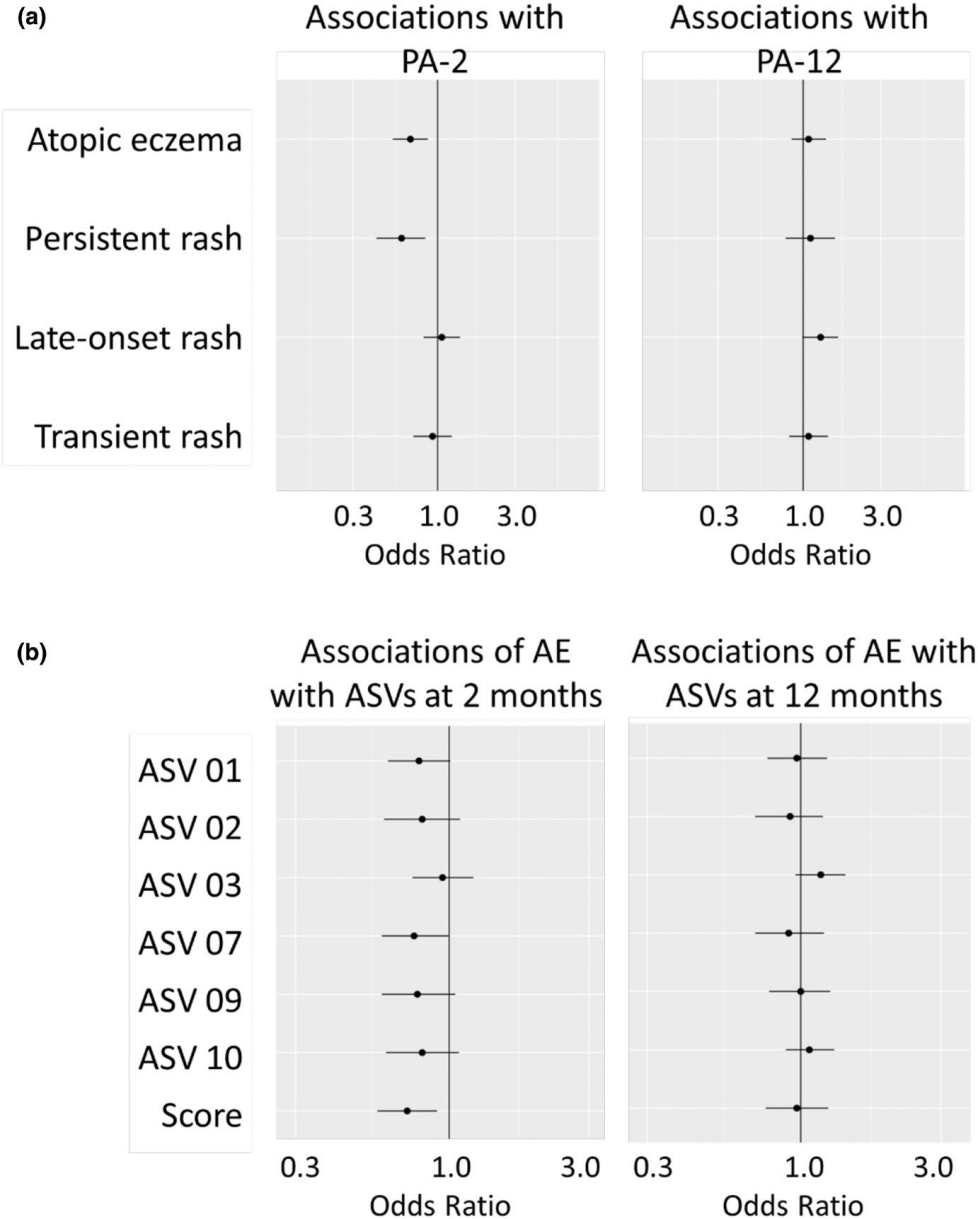
Atopic eczema and *Bifidobacterium* composition at 2 and 12 months. (A) Associations of atopic eczema and rash classes with primary *Bifidobacterium* axes at 2 (PA‐2) and 12 months (PA‐12). (B) Associations of atopic eczema (AE) with various *Bifidobacterium* ASVs and a summation score created from the relative abundance of the respective ASVs. For the description of ASVs see Table S1. *N* = 565.

PA‐2 was inversely related to AE (OR = 0.68 [0.53–0.87], Figure 3A) consistently in all centers (Figure S6). Likewise, PA‐2 was inversely related to persistent rash (OR = 0.60 [0.42–0.84], Figure 3A). When excluding children with AE diagnosed already at 2 months, the estimates hardly changed (OR 0.71 [0.56–0.91] and 0.60 [0.43–0.84], respectively). The strong association of PA‐2 with AE was independent from other measures of the intestinal microbiome at 2 months: α‐diversity and the abundance of other bacterial genera were not associated with AE (Table S3, Figure S7).

In contrast to 2 months, *Bifidobacterium* at 12 months (PA‐12) was not protective; rather, it was positively related to late‐onset rash (OR = 1.28 [0.99–1.64]). Excluding children with AE already diagnosed at 12 months slightly increased the estimate (OR = 1.34 [1.03–1.73]).

From this analysis it was not clear whether the opposite effects were caused by the ASV composition or by the time point. To disentangle these effects, we summed up the relative abundance of the main PA‐2 ASVs. This summation score was inversely related to AE (OR = 0.72 [0.57–0.91], Figure 3B). In contrast, there was no association when applying the same score to the 12 months samples (OR = 0.97 [0.76–1.24]). When building a summation score of the main PA‐12 ASVs, there was neither an association with AE (OR = 1.04 [0.82–1.32]) nor late‐onset rash (OR = 1.09 [0.86–1.37]). Sensitivity analyses with AE at 6 years supported these results (data not shown).

### 3.4 The composition of *Bifidobacterium* ASVs shifts from 2 to 12 months

**FIGURE 4:**
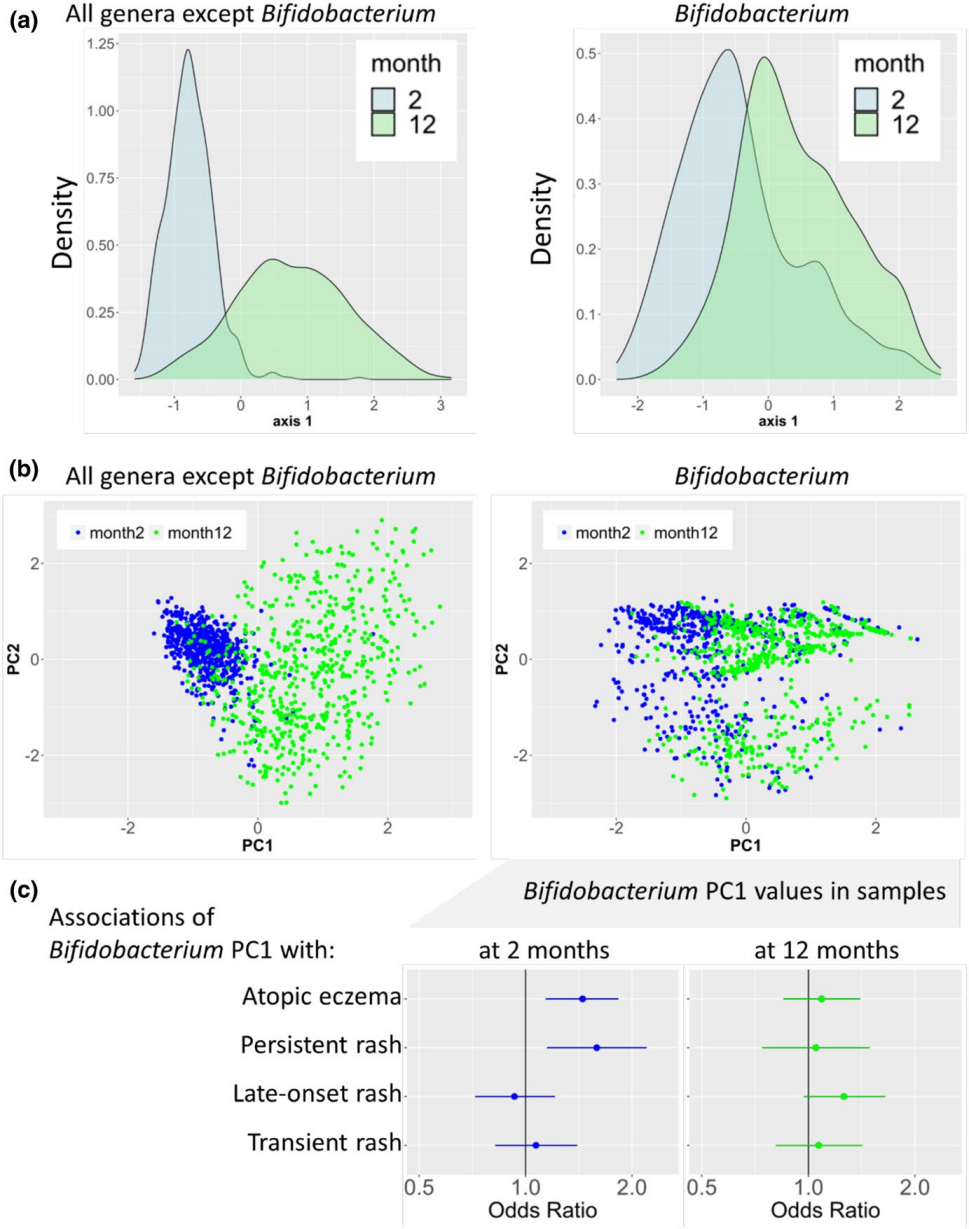
Common PCA over both time points (joint PCA). (A, B) Shown are density plots (A) and scatter plots (B) of the first (two) axes of the joint PCAs on ASVs unrelated (left) or related (right) to the genus *Bifidobacterium*. *N* = 618. (C) Associations of atopic eczema and rash classes with the first axis of the joint *Bifidobacterium* PCA restricted to samples taken at month 2 (left) or 12 (right). *N* = 565.

To better understand the shift from 2 to 12 months in the composition of the ASVs within the genus *Bifidobacterium*, we performed a further PCA including the *Bifidobacterium* ASVs from *both* time points, which we called the *joint* PCA. In analogy, we performed a joint PCA for all genera except *Bifidobacterium*. For the non‐*Bifidobacterium* ASVs, the first PCA axis revealed a clear separation of the samples from the two time points (Figure 4A,B, left panels). For *Bifidobacterium*, however, the distributions of the samples from the two respective time points overlapped considerably (Figure 4A,B, right panels). In samples taken at 2 months, high values of the first axis of the joint *Bifidobacterium* PCA (PA‐J) were positively associated with AE and persistent rash (Figure 4C, left panel), thereby suggesting detrimental effects of a premature loss of those ASVs that are typically found at 2 months. In samples taken at 12 months, however, PA‐J was unrelated to AE (Figure 4C, right panel).

**FIGURE 5:**
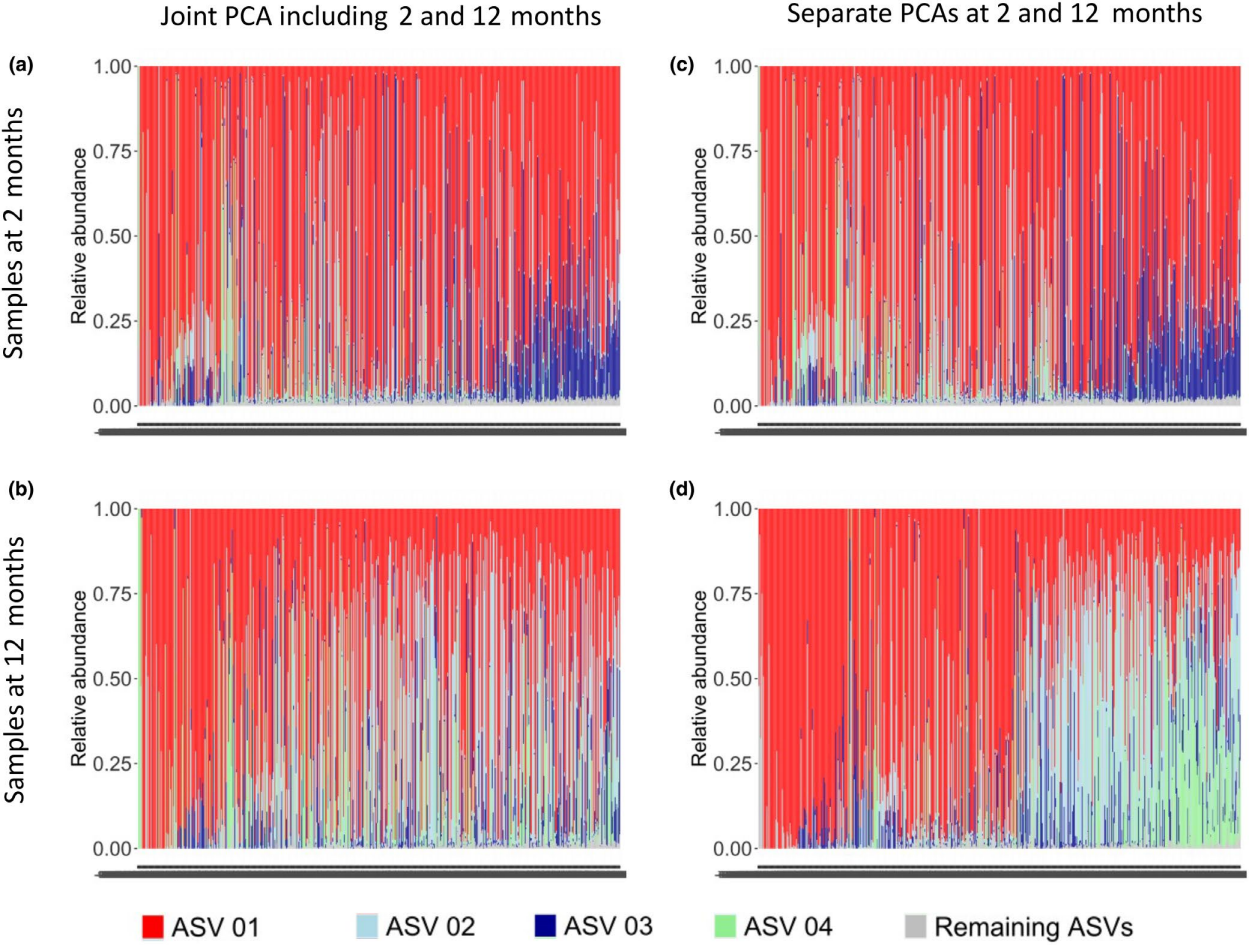
PCA over both time points versus separate time points. Barplots showing the composition of relative abundance of ASV01 (red), ASV02 (light blue), ASV03 (dark blue), ASV04 (green), and other ASVs (gray) per sample. Samples are sorted by increasing values of the respective first PCA axis. In (A), the inverted first PCA axis of the joint PCA is shown. *N* = 618.

When reverting the orientation of PA‐J, it strongly resembled PA‐2 as illustrated by a strong correlation (rho = 0.98) and a similar ASV composition (Figure 5A,C). In contrast, PA‐J was less strongly related to PA‐12 (rho = 0.75) and displayed dissimilar ASV patterns (Figure 5B,D).

### 3.5 Environmental influences on *Bifidobacterium* and their effects on atopic eczema

**FIGURE 6:**
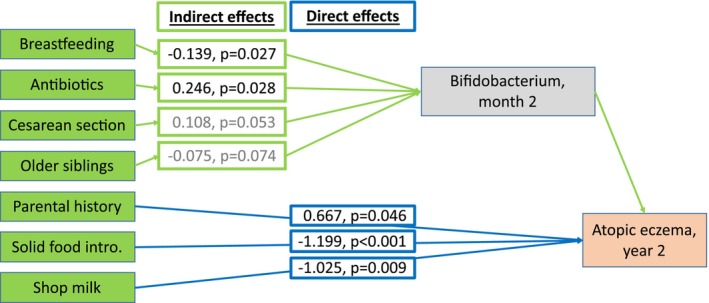
Mediation model showing the mediation by *Bifidobacterium*. Shown are beta estimates and *p*‐values for direct (blue) and indirect (green) effects with *p* < .1 in the mediation model. *N* = 565.

Breastfeeding and older siblings were positively related to *Bifidobacterium* at month 2, whereas antibiotics and cesarean section negatively impacted on *Bifidobacterium* at both time points (Figure S8). PA‐2 mediated preventive effects of breastfeeding and older siblings and risk effects of antibiotics and cesarean section on AE (Figure 6) and persistent rash (Figure S9). Independently of the gut microbiome, a parental history of AE was a direct risk factor for AE (Figure 6). Direct protective effects were seen for consumption of shop milk and for the introduction of solid foods during the first year of life.

## 4 DISCUSSION

In a comprehensive analysis of more than 600 children of a population‐based cohort, we found distinct variants of *Bifidobacterium* related to the pathogenesis of atopic eczema (AE). At 2 months, members of *Bifidobacterium* were dominant and showed the strongest and most consistent inverse associations with AE. Between 2 and 12 months, the members of this genus changed profoundly in composition on the ASV level. The newly emerging *Bifidobacterium* ASVs did not show protective effects, and also the previously beneficial ASVs lost their effects by 12 months. Effects of breastfeeding or antibiotics on AE were partly mediated by *Bifidobacterium* at month 2.

An AE diagnosis may cover several features, which may vary over time. Dissimilar definitions might explain the large range of prevalence estimates in epidemiological reports, for example, 12‐month diagnosis varying from 3% to 20% across countries.^35^ Here, we explored various aspects of AE using the full capacity of the longitudinal design of the PASTURE birth cohort. Because AE mostly manifests early in life, we chose a cumulative definition of parent report of doctor's diagnosis until 2 years. At 6 years, we used a simple question covering the entire time span to focus on disease manifestations that were still remembered after several years. Thus, we leveraged a phenomenon we observed in the context of asthma, where correct parental recall of wheezing episodes better reflected clinical relevance than episodes that were forgotten.^36^

To model disease trajectories, we employed a latent class analysis based on symptoms, similar to other studies.^4^, ^5^, ^6^, ^7^, ^8^, ^9^ We chose a 4‐class solution for consistency with previous reports.^4^, ^9^ The persistent class reflected an early emerging, more atopic, and clinically important phenotype, closely matching an AE diagnosis. Previous analyses of the PASTURE study demonstrated a strong association (OR = 17 [9–31]) of persistent rash with the scoring atopic dermatitis (SCORAD)‐score at 12 months thereby supporting the validity of our approach.^9^ The transient rash class, as a self‐limiting phenotype, may offer insight into secondary prevention. The late‐onset rash class may be a relatively mild phenotype that nevertheless affects individuals for years.

*Bifidobacterium* is among the first bacteria to colonize the human gut^37^ and they are dominant members of the intestinal microbial communities during breast‐feeding, typically at least for the first 3 to 4 months.^38^ This is seen also in our data, where at 2 months of age *Bifidobacterium* comprised about two‐thirds of all bacteria. These microorganisms are considered beneficial as reduced levels are associated with disease conditions.^38^, ^39^, ^40^, ^41^ In this context it is also notable that mere bacterial diversity, which is very commonly associated with health benefits, was not associated with AE during the first year of life.

Previous studies in young children report *Bifidobacterium* to be less common in AE patients, while in older children *Bifidobacterium* was more abundant in AE cases.^42^, ^43^, ^44^, ^45^ This discrepancy could point towards a temporal shift in the effects of *Bifidobacterium*. Likewise in our study, the ASV composition of *Bifidobacterium* at month 12 was associated with increased risk of late‐onset rash. Instead, other bacterial genera emerging during the first life might exert protective effects at 12 months, which was beyond the scope of this analysis.

While adequate maturation of the microbiome has been shown to protect against allergic diseases,^12^, ^13^ maturation too early in life might cause the opposite.^12^, ^46^ In our study, this notion is reflected by the increased risk of AE due to a premature shift towards an adult‐like *Bifidobacterium* composition. In this regard, AE might be distinct from asthma where maturation of *Bifidobacterium* and other genera beyond 2 months was more relevant for disease.^12^

Age‐related changes within bifidobacterial components are well known.^47^ *Bifidobacterium longum*, *B. breve*, and *B. bifidum* are generally dominant in infants, whereas *B. catenulatum* and *B. adolescentis* are more prevalent in adults.^39^ In our samples, the adult‐like group, especially *B. adolescentis*, was already present at 12 months. In a sensitivity analysis, we used a combination of ASVs relevant to protection at month 2 and applied it to the 12‐month samples but the combination no longer showed a protective effect. This phenomenon suggests that protection against AE by *Bifidobacterium* depends on a critical window and an adequate combination of species. Likewise, studies on species level suggested a higher prevalence of *B. adolescentis* and a lower prevalence of *B. bifidum* in infants with AE as compared to healthy infants.^48^, ^49^

Many *Bifidobacterium* strains used in dietary probiotics belong to the “early” species.^38^ As shown here, not all ASVs of a species exert the same effects. This may explain why the effectiveness of probiotics for AE is inconsistent.^50^, ^51^, ^52^ Though successful prevention by probiotics have been reported for AE,^53^ a meta‐analysis including 39 randomized controlled trials involving 2599 participants aged from 0 to 55 years did not identify a clear beneficial effect on AE symptoms.^50^ Probiotics may fail to successfully compete with established commensals.^54^ In addition, the window of opportunity, which might extend to prenatal exposure,^55^ might have been missed in some studies.

The main determinants of early *Bifidobacterium* in our analysis were breastfeeding, antibiotics, and vaginal delivery, which is consistent with previous reports.^56^, ^57^, ^58^ In our study, early breastfeeding protected from AE by fostering *Bifidobacterium*. Infant‐type *Bifidobacterium* species such as *bifidum*, *longum subsp. infantis*, and *breve* share a specific ability to digest human milk oligosaccharides (HMO) in breastmilk, thereby profiting from a strong selective advantage.^38^ The most common species in our study at 2 months were *B. longum* and *B. bifidum*, which are producers of beneficial aromatic lactic acids.^59^

A major strength of our study is the large sample size, particularly when compared to previous studies on gut microbiota and AE. In addition, we have covered the most important period of microbial development, that is, the first year of life. Furthermore, we used multiple outcome definitions to capture the various aspects of AE in childhood.

Our study is limited in that ASVs could not be classified on a species level with appropriate reliability due to insufficient sequence length. However, we were able to draw a sufficiently confident picture of infant and adult‐type bifidobacterial composition utilizing an exploratory combined assessment of BLAST and TRFLP data. Here we focused on bifidobacterial subgenus composition, but we acknowledge that other bacterial genera changed over time (Figure 1), which might stimulate future research. Although selection of children in rural areas might reduce generalizability of findings, farm‐children studies have previously been considered informative with respect to environmental effects on immune‐mediated diseases.

Observational studies cannot establish causality; therefore, our usage of a “protective effect” is conditional on future interventions, which need to consider an adequate ASV composition particularly very early in life.

Taken together, our findings highlight the importance of an adequately timed maturation of the gut microbiota during the first year of life. In particular, *Bifidobacterium* showed a Janus face with protective and risk effects determined by their maturational stage in terms of ASV composition and time point of appearance. In the future, a disease‐prone gut microbiome might be reset by a more targeted approach considering adequate understanding of the important bacterial strains and critical windows for intervention.

## CONFLICT OF INTEREST STATEMENT

Amandine Divaret‐Chauveau reports grants from Don du Souffle, from Fondation du Souffle, from ARAIRLOR (Association Régionale d'Aide aux Insuffisants Respiratoires de Lorraine), from French public agency ASES, and from Novartis; received consulting fees from Sanofi, Stallergens, ALK and Aimmune Therpeutics; received honoraria for lectures and travel support for attending meetings from Novartis and ALK, and has stock options of Essilor Luxottica. Bianca Schaub reports consulting fees from GlaxoSmithKline, Novartis, ALK, Astra Zeneca and Sanofi. Markus Ege is an employee inventor of patents EP000002361632B1, EP000001964570B1, US000009950017B2. His employer has received investigational milk products from FrieslandCampina. EvM reports funding from the European Commission (LSH‐2004‐1.2.5‐1‐018996), the European Research Council (ERC‐2009‐AdG), Deutsche Forschungsgemeinschaft (Leibniz Price 2013), German Federal Ministry of Education and Research (German Center for Lung Research – DZL, Go Bio Initial Grant), Bavarian State Ministry of Health and Care for “URS Study”, OM Pharma S.A. (“Impact Chip Study”, “BEAR Study”); royalties from Elsevier GmbH, Georg Thieme Verlag, Springer‐Verlag GmbH, Elsevier Ltd., Springer Nature Group, Deutscher Apotheker Verlag, consulting fees from the Chinese University of Hongkong, European Commission, AstraZeneca, Imperial College London, OM Pharma S.A.; honoraria from ALK‐Abello Arzneimittel GmbH, Japanese Society of Pediatric Allergy and Clinical Immunology (JSPACI), Klinikum Rechts der Isar, University of Colorado, Paul‐Martini‐Stiftung, Astra Zeneca BioPharmaceuticals Medical, Imperial College London, Children’s Hospital Research Institute of Manitoba Kompetenzzentrum für Ernährung (Kern), OM Pharma S.A., Swedish Pediatric Society for Allergy and Lung Medicine, Chinese College of Allergy and Asthma (CCAA), Abbott Laboratories, Deutscher Apotheker Verlag GmbH & Co. KG, Socieded Chilena de Enfermedades Respiratorias, Japanese Society of Allergology, British Society for Asthma and Clinical Immunology, American Academy of Allergy, Asthma & Immunology, European Respiratory Society (ERS); support for attending meetings from Deutsches Zentrum für Lungenforschung (DZL), Fabio Luigi Massimo Ricciardolo/Contatto S.r.l., Fraunhofer ITEM Hannover, MCCA Institut für Immunologie Uni Wien, Karl‐Landsteiner Privatuniversität f. Gesundheitswissenschaften, Swiss Institute of Allergy and Asthma Research (SIAF) Davos (Associated Institute of the University of Zurich) MHH (Medizinische Hochschule Hannover), Natasha Allergy Research Foundation, Deutsche Forschungsgemeinschaft, Gordon Research Conferences, Socieded Chilena de Enfermedades Respiratorias, Arla, Universität Leiden, OM Pharma S.A., American Academy of Allergy, Asthma & Clinical Immunology, Deutsche Forschungsgemeinschaft (DFG), European Respiratory Society (ERS), Deutsche Gesellschaft für Kinder‐ und Jugendmedizin, World Allergy Organization (WAO), European Parliament, Gesellschaft für Pädiatrische Pneumologie (GPP), Helmholtz Association of German Research Centres, International Balzan Foundation "Prize". EvM has patent No. PCT/EP2019/085016 (Barn dust extract for the prevention and treatment of diseases) pending (Barn dust extract for the prevention and treatment of diseases) pending, royalties paid to ProtectImmun for patent EP2361632 (Speciﬁc environmental bacteria for the protection from and/or the treatment of allergic, chronic inﬂammatory and/or autoimmune disorders, granted on 19 March 2014), and patents EP1411977 (Composition containing bacterial antigens used for the prophylaxis and the treatment of allergic diseases, granted on 18 April 2007), EP1637147 (Stable dust extract for allergy protection, granted on 10 December 2008), and EP 1964570 (Pharmaceutical compound to protect against allergies and inﬂammatory diseases, granted on 21 November 2012) licensed to ProtectImmun., Patent EP21189353.2. 2021. von Mutius E, Rankl B, Bracher F, Müller C, Walker A, Hauck SM, Merl‐Pham J, inventors; PROTEINS IDENTIFIED FROM BARN DUST EXTRACT FOR THE PREVENTION AND TREATMENT OF DISEASES, Patent PCT/US2021/016918. 2021. Martinez FD, Vercelli D, Snyder SA, von Mutius E, Pivniouk V, Marques dos Santos M, inventors; THERAPEUTIC FRACTIONS AND PROTEINS FROM ASTHMA‐PROTECTIVE FARM DUST, Patent EP21189353.2. 2021. von Mutius E, Rankl B, Bracher F, Müller C, Walker A, Hauck SM, Merl‐Pham J, Adler H, Yildirim A.Ö., Sattler M, Santos Dias Mourao A, Borggräfe J, O´Connor P.D., Plettenburg O, inventors; PROTEINS IDENTIFIED FROM BARN DUST EXTRACT FOR THE PREVENTION AND TREATMENT OF DISEASES. EvM has been serving as Member of the EXPANSE (funded by European Commission) Scientific Advisory Board, Member of the BEAMS External Scientific Advisory Board (ESAB), Member of the Editorial Board of “The Journal of Allergy and Clinical Immunology: In Practice”, Member of the Scientific Advisory Board of the Children’s Respiratory and Environmental Workgroup (CREW), Member of the International Scientific & Societal Advisory Board (ISSAB) of Utrecht Life Sciences (ULS) University of Utrecht, Member of External Review Panel of the Faculty of Veterinary Science, University of Utrecht, Member of the Selection Committee for the Gottfried Wilhelm Leibniz Programme (DFG), Member of the International Advisory Board of Asthma UK Centre for Applied Research (AUKCAR), Member of the International Advisory Board of “The Lancet Respiratory Medicine”, Member of the Scientific Advisory Board of the CHILD (Canadian Healthy Infant Longitudinal Development) study, McMaster University, Hamilton, Canada, Asthma UK Centre for Applied Research, Pediatric Scientific Advisory Board Iceland, Abbott Allergy Risk Reduction Advisory Board.

## Supporting information


Appendix S1.

## Data Availability

The raw DNA sequence data, that is, the demultiplexed 16S rRNA amplicon reads have been deposited to NCBI Sequence Read Archive under the accession numbers PRJNA1068358 and PRJNA1068358 for 2‐ and 12‐month data, respectively. Upon request the metadata will be shared on an aggregate level. The PASTURE study is an ongoing cohort with fieldwork still taking place. Therefore, data cannot be anonymized. According to European law, health‐related data can only be shared on an aggregate level in order to avoid identification of individuals.

## Appendix

The members of the PASTURE Study Group are (in Alphabetical Order by Study Center): Täubel, M. (Finland); Dalphin, ML.; Laurent, L. (France); Beerweiler, C.; Böck, A.; Foppiano, F.; Hose AJ., Illi S.; Pechlivanis, S.; Theodorou, J. (Germany); Frei, R. (Switzerland).
