# Supplementary material for: The Janus face of Bifidobacterium in the development of atopic eczema: A role for compositional maturation
Source: Pediatr Allergy Immunol. 2025 Feb 11;36(2):e70041. doi: 10.1111/pai.70041 (PMC11812080; doi:10.1111/pai.70041)
Supplement: Supplementary file 1 — Appendix S1. [file PAI-36-e70041-s001.docx]

**The Janus face of Bifidobacterium in the development of atopic eczema: a role for compositional maturation**

Martin Depner, PhD,^1^ Diana Hazard Taft, PhD,^2^ Stefanie Peschel, Msc,^1^ Caroline Roduit, MD, PhD,^3,4,5^ Anne M. Karvonen, PhD,^6^ Cindy Barnig, MD, PhD^7,8^ Amandine Divaret-Chauveau, MD,^9,10.11^ Josef Riedler, MD,^12^ Juha Pekkanen, MD, PhD,^6,13^ Elisabeth Schmausser-Hechfellner, BSc,^1^ Giulia Pagani, PhD,^1^ Roger Lauener, MD,^3, 4,14,15^ Marjut Roponen, PhD,^16^ Harald Renz, MD, PhD,^17,18^ Petra Ina Pfefferle, PhD, DrPH^18,19^ Bianca Schaub, MD^18,20^ Erika von Mutius, MD, MSc^1,18,20^ Pirkka V. Kirjavainen, PhD,^6,21,*^ Markus J. Ege, MD, MPH^1,18,20,*^ and the PASTURE study group^22^

##### AFFILIATIONS:

^1^ Institute of Asthma and Allergy Prevention, Helmholtz Zentrum Munich, German Research Center for Environmental Health, Neuherberg, Germany

^2^ Food Science and Technology, University of California, Davis, USA

^3^ Christine Kühne Center for Allergy Research and Education (CK-CARE), Davos, Switzerland

^4^ Children’s Hospital of Eastern Switzerland, St. Gallen, Switzerland

^5^ Division of Respiratory Medicine and Allergology, Department of Paediatrics, Inselspital, University of Bern, Bern, Switzerland

^6^ Department of Health Security, National Institute for Health and Welfare, Kuopio, Finland

^7^ Department of Respiratory Disease, University Hospital of Besançon, Besançon, France.

^8^ INSERM, EFS BFC, UMR1098, Interactions Hôte-Greffon-Tumeur/Ingénierie Cellulaire et Génique, University of Franche-Comté, Besançon, France.

^9^ Pediatric Allergy Department, Children’s Hospital, University Hospital of Nancy, Vandoeuvre les Nancy, France

^10^ EA 3450 DevAH, Faculty of Medecine, University of Lorraine, Vandoeuvre les Nancy, France

^11^ UMR/CNRS 6249 Chrono-environnement, University of Bourgogne Franche-Comté, Besançon, France

^12^ Children’s Hospital Schwarzach, Schwarzach, Austria

^13^ Department of Public Health, University of Helsinki, Helsinki, Finland

^14^ University of Zurich, Zurich, Switzerland

^15^ School of Medicine, University of St Gallen, St Gallen, Switzerland

^16^ Department of Environmental and Biological Sciences, University of Eastern Finland, Kuopio, Finland

^17^ Institute for Medicine Laboratory, Pathobiochemistry and Molecular Diagnostics, Philipps-University Marburg, Marburg, Germany.

^18^ German Center for Lung Research (DZL)

^19^ Comprehensive Biobank Marburg (CBBMR), Philipps-University Marburg, Marburg, Germany

^20^ Dr. von Hauner Children’s Hospital, Ludwig Maximilians University Munich, Munich, Germany

^21^ Institute of Public Health and Clinical Nutrition, University of Eastern Finland, Kuopio, Finland

^22^ The members of the PASTURE study group are (in alphabetical order by study center): Täubel, M. (Finland); Dalphin, ML.; Laurent, L. (France); Beerweiler, C.; Böck, A.; Foppiano, F.; Hose AJ., Illi S.; Pechlivanis, S.; Theodorou, J. (Germany); Frei, R. (Switzerland)

* contributed equally

Address for correspondence:

Prof. Dr. med. Markus Ege, MPH

Dr von Hauner Children’s Hospital, LMU Munich

Lindwurmstr. 4, 88337 Munich, Germany

Email: markus.ege@med.lmu.de

# Supplemental Methods

## Assessment of exposures

The children were recruited at birth and seen at 2, 12, 54, and 72 months during home or clinic visits, and parents completed questionnaires at 2, 12, 18, 24, 36, 48, 60 and 72 months.^1^ Beside the mode of delivery (Cesarean section vs. vaginal delivery) potential environmental or nutritional determinants of eczema as well as medication were assessed. Data on treatment with systemic antibiotics was available for the first 2 months and the first year beyond two months (between month 2 and month 12). Likewise, the mother’s intake of antibiotics during pregnancy or during breastfeeding was investigated. Breastfeeding was defined as non-exclusive breastfeeding at month 2; duration of non-exclusive breastfeeding was dichotomized at 6 months.

Solid food introduction was defined as the introduction of six main food items in course of the first year of life, namely cereals, meat, bread, yogurt, cake, and vegetables or fruits as previously described.^2^ Furthermore, the children’s diet was assessed with respect to the type of supplemental food and its introduction in terms of at least weekly consumption.^3^ Introduction of specific nutrition includes shop milk or farm milk in the first year of life. Farm milk consumption was defined as weekly consumption of any milk obtained directly from a farm irrespectively of boiling or skimming. Farm exposure was defined as growing up on a family run farm. Among family-related variables, we assessed the number of siblings (at least two older siblings) and parental history of eczema, hay fever, and asthma.

## DNA-extraction of fecal samples

Fecal samples were frozen within 10 minutes from collection and stored at -20°C until further processing.^4^ At a central laboratory (THL Kuopio, Finland), DNA was extracted from the fecal samples in batches as follows: Partially defrosted fecal samples were homogenized using Stomacher® 80 micro-Biomaster (Seward Ltd, UK) laboratory paddle blender (2 min at high speed). DNA was extracted from 150 mg of ice-cold homogenized fecal sample, using bead-beating method with Zymo Research fecal DNA MiniPrep™ kit (Catalog No. D6010, Zymo Research, Irvine, CA) according to the manufacturer’s instructions. The bead-beating step was done using FastPrep® FP120 homogenizer (2 min at full speed 6.5 m/s). Finally, the samples were eluted with 100 µL of elution buffer D3004-4-10 (Zymo Research, Freiburg, Germany). The sample extracts were kept on ice throughout the entire procedure. The extracted DNA was immediately frozen at -20°C and stored at -80°C.

## Sequencing analyses

Amplification and sequencing of the V4 region of the 16S rRNA gene were performed as described previously,^5-7^ using primers F515 (5′–NNNNNNNNGTGTGCCAGCMGCCGCGGTAA–3′) and R806 (5′–GGACTACHVGGGTWTCTAAT–3′).^8^ Samples were amplified in triplicate PCR reactions. Triplicate reactions were pooled and run on agarose gel to confirm amplification. Samples were then pooled for inclusion in sequencing runs and cleaned using the QiaQuick PCR purification kit. The pooled samples were submitted to the DNA Technologies Core at the University of California Davis Genome Center for Illumina paired-end library preparation with blunt-end addition of sequencing adaptors, and sequencing using 250-bp paired-end chemistry on an Illumina MiSeq instrument in multiple runs.

### Data processing

Raw sequencing data from each run were demultiplexed, imported into QIIME2-2018.6 and quality trimmed. Reads were denoised using DADA2^9^ as implemented in QIIME2.^10^ Taxonomy was assigned to representative sequences using a Naive Bayes classifier trained on the Silva 138 99% database specific to the 515F–806R region (updated version available at https://docs.qiime2.org/2024.10/). The current taxonomic assignment was largely consistent with previous assignments based on Greengenes (Figure S1). Contaminant sequences were removed using the R package decontam.^11^ After the exclusion of amplicon sequence variants (ASVs) representing eukaryotes, archaea, cyanobacteria, mitochondria, or unassigned kingdom, 5,834 ASVs remained.

*Bifidobacterium* specific Terminal Restriction Fragment Length Polymorphism (TRFLP) was performed as previously described.^12^ For suggestive identification of ASV-sequences, we used the basic local assignment tool (BLAST) with 16S ribosomal RNA database limited to sequences from type material (https://blast.ncbi.nlm.nih.gov). We report all unique species with 100% query cover and 100% identity; if 100% identity was not achieved, we report the best match. The identity of bifidobacterial species was approximated by both the BLAST search and the TRFLP-analysis.

### Statistical analysis

Statistical analysis was performed with R 4.3.1,^13^ particularly with the phyloseq package.^14^ Upon request, computer code will be made available to readers. Latent class analysis (LCA) on rash from 1 to 6 years was performed using R-package poLCA^15^ after imputation of the respective missing rash values with the mice package in R.^16^

Rarefaction and calculation of species richness and Shannon’s diversity index was iterated 1000 times and the resulting measures of alpha-diversity were subsequently averaged.

Similar to the eigengene analysis,^17^ we used the first axes of principal component analyses (PCA) on the *Bifidobacterium* ASVs at months 2 and 12, respectively. The procedure was done for month 2 and month 12 separately. The first PCA axes were oriented to positively correlate with the relative abundance of the *Bifidobacterium* genus. For other genera we performed analogous PCAs.

In a joint PCA, ASVs of month 2 and month 12 of the genus *Bifidobacterium* were entered in the same PCA. After having defined the axes, samples of month 2 and month 12 were analyzed separately. For all other genera except *Bifidobacterium*, we calculated a joint PCA over both time points in analogy.

Regression models were used to find associations between microbial variables and the outcomes or the environmental determinants. To compare indirect and direct effects, mediation models were calculated in Mplus.^18^ Potential determinants were cesarean section, antibiotics, presence of two older siblings, mode of delivery, parental history, introduction of solid food, consumption of farm milk and shop milk, growing up on a farm, and contact to farm animals in pregnancy.

Network analyses were carried out using the R package NetCoMi (v1.0.3).^19^ Correlations between bacterial genera in networks were estimated using the SparCC approach,^20^ while elsewhere the Spearman coefficient was used as measure of correlation.

P-values < 0.5 were considered significant and always based on two-sided tests. Because of the correlation within exposures and within outcomes correction for multiple testing was not useful.

# Supplemental Tables

**Table S1: Characteristics of the study population stratified by atopic eczema (AE)**

| **Characteristics** | **Complete data** | **Without AE (n=481)** | | **With AE (n=84)** | |  |
| --- | --- | --- | --- | --- | --- | --- |
|  | n | n | % | n | % | p-value |
| Male gender | 565 | 236 | 49.1 | 35 | 41.7 | 0.257 |
| Austria | 565 | 111 | 23.1 | 22 | 26.2 | 0.630 |
| Switzerland | 565 | 171 | 35.6 | 16 | 19.0 | 0.005 |
| Germany | 565 | 115 | 23.9 | 16 | 19.0 | 0.404 |
| Finland | 565 | 84 | 17.5 | 30 | 35.7 | 0.000 |
| Cesarean section | 563 | 84 | 17.5 | 13 | 15.5 | 0.761 |
| Antibiotics (first 2 months) | 564 | 394 | 4.2 | 67 | 4.8 | 1.000 |
| Breastfeeding at month 2 | 565 | 20 | 81.9 | 4 | 80.7 | 0.916 |
| Consumption of farm milk | 557 | 130 | 27.4 | 12 | 14.5 | 0.018 |
| Consumption of shop milk | 557 | 123 | 25.9 | 10 | 12.0 | 0.009 |
| All 6 main food items within 12 months | 561 | 340 | 71.1 | 36 | 43.4 | 0.000 |
| Environmental smoke (first two months) | 564 | 19 | 4.0 | 3 | 3.6 | 1.000 |
| Growing up on a farm | 565 | 228 | 47.4 | 40 | 47.6 | 1.000 |
| At least two older siblings | 565 | 160 | 33.3 | 33 | 39.3 | 0.343 |
| Parental education high | 563 | 434 | 90.6 | 77 | 91.7 | 0.916 |
| Parental atopic eczema | 561 | 52 | 10.9 | 20 | 24.1 | 0.002 |
| Parental asthma | 560 | 51 | 10.7 | 15 | 18.1 | 0.082 |
| Parental hayfever | 564 | 128 | 26.7 | 30 | 35.7 | 0.116 |
| Contact to farm animals in pregnancy | 560 | 331 | 69.5 | 47 | 56.0 | 0.020 |
|  |  |  | mean |  | mean |  |
| Birthweight (g) |  |  | 3513 |  | 3517 | 0.941 |
| Exclusive breastfeeding (months) |  |  | 3.37 |  | 3.07 | 0.301 |
| Any breastfeeding (months) |  |  | 7.35 |  | 7.13 | 0.694 |

Table S2: Description of the main *Bifidobacterium* ASVs

| **ASV** | **32 digit code** | **Mean relative abundance**  **in Bifidobacterium** | | | **Summation Score^§^** | | **Designated**  **names** | **BLAST results with 100% identity** |
| --- | --- | --- | --- | --- | --- | --- | --- | --- |
|  |  | all samples | month 2 | month 12 | month 2 | month 12 |  |  |
| ASV_01 | 69e611251f4d8582e312afa5737f033e | **62.83** | 68.42 | 57.22 | 0.43 |  | *"longum 1"* | *Bifidobacterium cebidarum, colobi, castoris, samirii,*  *canis, longum, pseudolongum, breve, choerinum* |
| ASV_02 | 8347bd34436f72573fcde614b95d4702 | **15.79** | 10.79 | 20.81 | 0.56 | 0.77 | *"pseudoca tenulatum 1"* | *Bifidobacterium miconisargentati, callitrichidarum, apri,*  *merycicum, gallicum, catenulatum, pseudocatenulatum,*  *angulatum* |
| ASV_03 | 7b223719a0af567e7ea99f06f7ea1068 | **9.40** | 11.52 | 7.28 | 0.77 | 0.55 | *"bifidum 1"* | *Bifidobacterium bifidum* |
| ASV_04 | 554c761996ebab999befda1b695fd81d | **7.18** | 4.14 | 10.23 |  | 0.58 | *"adolescentis 1"* | *Bifidobacterium adolescentis, faecale* |
| ASV_05 | 5d564d5db4ddb67c61b7fdfbffbe15d3 | **1.99** | 2.73 | 1.26 |  |  | *"dentium 1"* | *B. dentium, B. moukalabense* |
| ASV_06 | 6fd9843eb698930e30818d77d505ed5c | **0.89** | 0.27 | 1.50 |  |  | *"animalis"* | *B. animalis* |
| ASV_07 | a5b58779401c242ca6754aa373d3744b | **0.53** | 0.78 | 0.27 | 0.50 |  | *"longum 2"* | *Bifidobacterium cebidarum, colobi, castoris, samirii,*  *canis, longum, pseudolongum, breve, choerinum,* |
| ASV_08 | 96be8572ab1e63d07eae18c99c131814 | **0.12** | 0.12 | 0.13 |  | 0.49 | *"pseudoca- tenulatum 2"* | *Bifidobacterium miconisargentati, callitrichidarum, apri,*  *merycicum, gallicum, catenulatum, pseudocatenulatum,*  *angulatum* |
| ASV_09 | 4b1fb308dd4f6b665da5b326030b59b1 | **0.11** | 0.20 | 0.03 | 0.53 |  | *"longum 3"* | *Bifidobacterium cebidarum, colobi, castoris, samirii,*  *canis, longum, pseudolongum, breve, choerinum,* |
| ASV_10 | f6be02442c9b4f55b808ad8cafa5a157 | **0.07** | 0.12 | 0.02 | 0.67 | 0.28 | *"bifidum 2"* | *Bifidobacterium bifidum* |
| ASV_11 | eb9076c8e44bbb76c4b75bba54206557 | **0.03** | 0.03 | 0.03 |  | 0.32 | *"adolescentis 2"* | *Bifidobacterium adolescentis, faecale* |
| ASV_12 | 27bec72984c7b8ce549a1bc31ceda5b9 | **0.02** | 0.04 | 0.01 |  |  | *"pseudoca- tenulatum 3"* | None (99.6% B*.callitrichidarum, B. gallicum,*  *B. catenulatum, B. pseudocatenulatum*) |
| ASV_13 | 2900b24a601ca841428836f424927d4d | **0.01** | 0.02 | 0.01 |  |  | *"dentium 2"* | *B. dentium, B. moukalabense, B. stellenboschense* |

^§^ Summation score using taxa substantially correlated (ρ>0.2) with the first PCA-axis at 2 and 12 months, respectively. Numbers are Spearman-correlations of the relative abundance of the respective ASVs with the corresponding first PCA-axis.

**Table S3: Associations of atopic eczema with measures of bacterial α-diversity**

| Time point | Measure of α-diversity | Odds Ratio  (Confidence-Interval) |
| --- | --- | --- |
| Month 2 | Richness | 0.91 [0.71-1.17] |
|  | Shannon | 1.00 [0.77-1.28] |
| Month 12 | Richness | 0.88 [0.69-1.12] |
|  | Shannon | 0.86 [0.68-1.09] |

# Supplemental figures

Figure S1: Reassignment of taxonomy


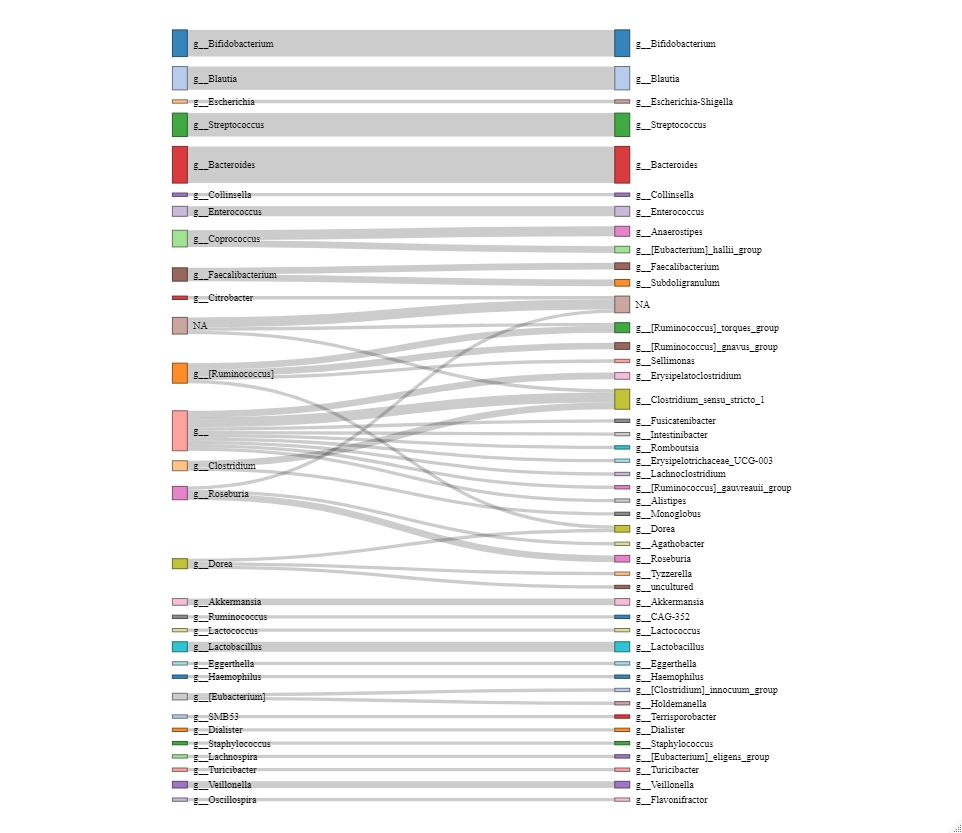


Changes in the classification of the genera from Greengenes to Silva 132. The figure is restricted to the genera represented by the 100 most frequent ASVs.

Figure S2: Participant flow

AE=atopic eczema, NA= not available (missing values), AT=Austria, CH=Switzerland, DE=Germany, SF=Finland, FR=France

By design, fecal sampling at month 2 was not performed in the French arm; therefore, these children were not included in the current analyses. Of the 930 children of the other four centers, 618 had data for both time points, i.e., month 2 and month 12.

Figure S3: Patterns of itchy rash according latent class analysis


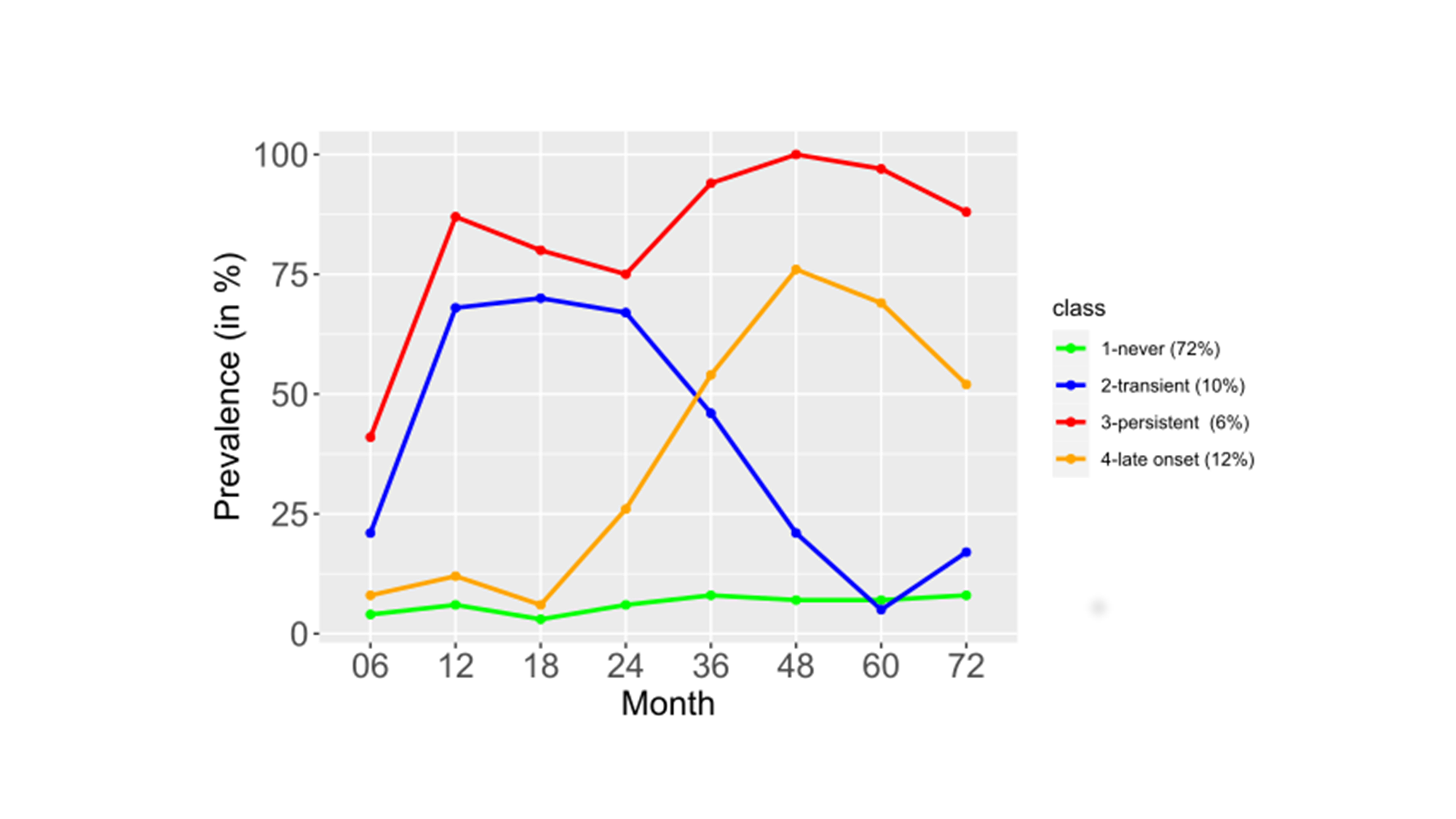


Prevalence of eczema symptoms, i.e., itchy rash, is plotted for each individual latent class against age in months. N=618. The class “never” was consistently used as a reference, when assessing the effects of the classes transient, persistent, and late onset. The two other classes not involved in the assessment were put on missing values for the respective models.

Figure S4: Correlation of primary PCA axes with relative abundance of *Bifidobacterium* ASVs

Spearman correlations are given for individual amplicon sequence variants (ASV) and the first Bifidobacterium PCA axes at 2 and 12 months, respectively. The ASV are named by the first 6 digits of their 32-digit code. The most common ASVs were renamed by a numerical 2-digit code for a straightforward presentation of subsequent analyses as explained in Table S2.

Figure S5: Correlation network of individual ASVs.


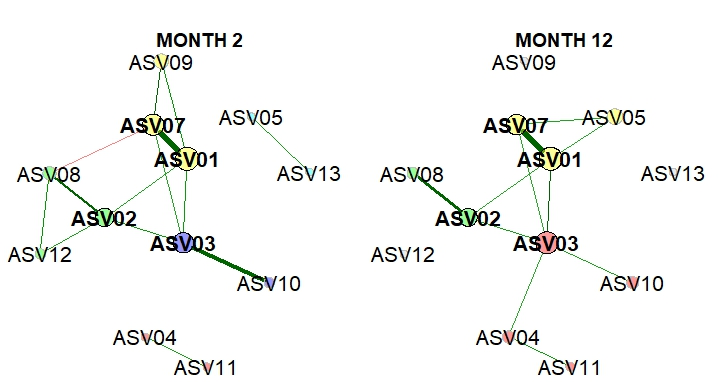


Green and red edges represent positive and negative correlations, respectively. Edge thickness indicates correlation strength. Only edges corresponding to correlations with a magnitude greater than 0.2 are drawn. Node sizes reflect the relative abundances of the ASVs. Clusters determined using greedy modularity optimization are represented by node colors. N=618

Figure S6: Consistency of effect of Bifidobacteria on atopic eczema

| Month 2 | Month 12 |
| --- | --- |
| 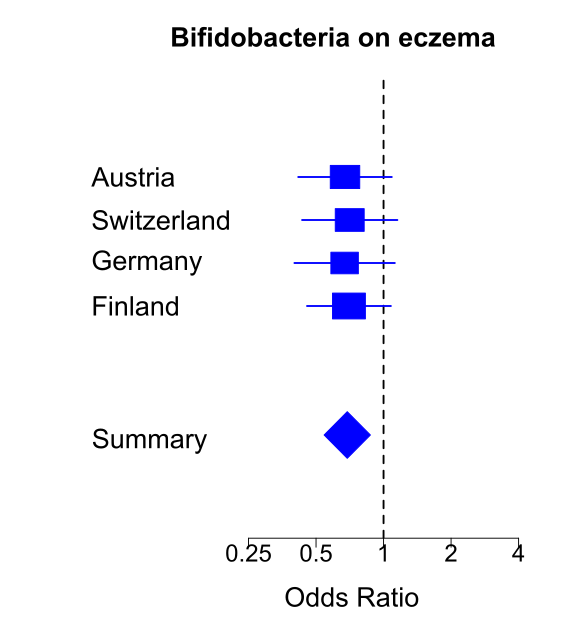 | 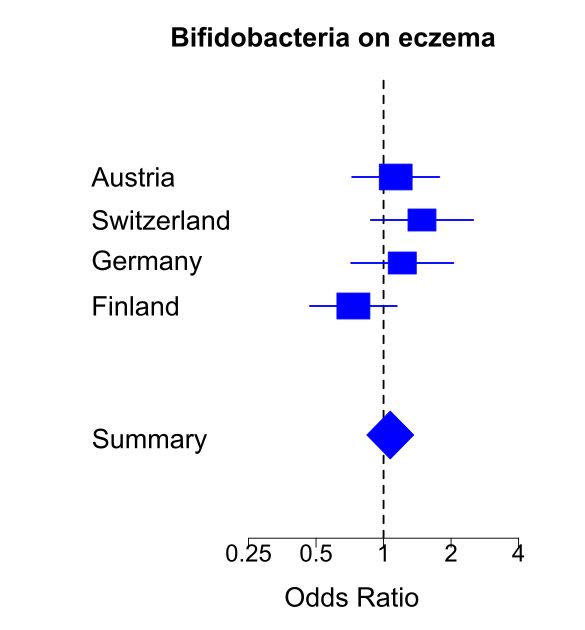 |

Meta-analysis of the effect of the genus *Bifidobacterium* (PCA axes at 2 and at 12 months) on atopic eczema. N=565

Figure S7: Associations of atopic eczema with bacterial genera at 2 months


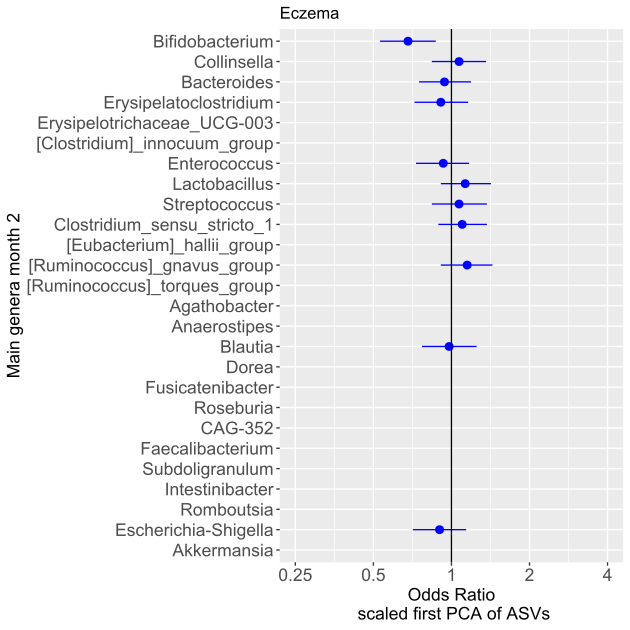


Associations are only shown for the main genera (mean relative abundance >=0.5%). All models are adjusted for study center. N=565

Figure S8: Determinants of PCA axes of *Bifidobacterium*

Month 2 Month 12

Bivariate models for significant (p<0.05) determinants are shown. Variables remaining in final models after stepwise selection are colored in red. All models are adjusted for study center. N=618

Figure S9: Mediation model for persistent rash

Shown are beta estimates and p-values for direct (blue) and indirect effects (green) with p<0.1 in the mediation model.

N=482

# REFERENCES

1. Loss GJ, Depner M, Hose AJ, Genuneit J, Karvonen AM, Hyvarinen A, et al. The Early Development of Wheeze. Environmental Determinants and Genetic Susceptibility at 17q21. Am J Respir Crit Care Med. 2016;193(8):889-97.

2. Roduit C, Frei R, Depner M, Schaub B, Loss G, Genuneit J, et al. Increased food diversity in the first year of life is inversely associated with allergic diseases. J Allergy Clin Immunol. 2014;133(4):1056-64.

3. Depner M, Ege MJ, Genuneit J, Pekkanen J, Roponen M, Hirvonen MR, et al. Atopic sensitization in the first year of life. J Allergy Clin Immunol. 2013;131(3):781-8.

4. Depner M, Taft DH, Kirjavainen PV, Kalanetra KM, Karvonen AM, Peschel S, et al. Maturation of the gut microbiome during the first year of life contributes to the protective farm effect on childhood asthma. Nature medicine. 2020;26(11):1766-75.

5. Bokulich NA, Mills DA. Facility-specific "house" microbiome drives microbial landscapes of artisan cheesemaking plants. Appl Environ Microbiol. 2013;79(17):5214-23.

6. Bokulich NA, Thorngate JH, Richardson PM, Mills DA. Microbial biogeography of wine grapes is conditioned by cultivar, vintage, and climate. Proceedings of the National Academy of Sciences of the United States of America. 2014;111(1):E139-48.

7. Bokulich NA, Mills DA. Improved selection of internal transcribed spacer-specific primers enables quantitative, ultra-high-throughput profiling of fungal communities. Appl Environ Microbiol. 2013;79(8):2519-26.

8. Caporaso JG, Lauber CL, Walters WA, Berg-Lyons D, Lozupone CA, Turnbaugh PJ, et al. Global patterns of 16S rRNA diversity at a depth of millions of sequences per sample. Proceedings of the National Academy of Sciences of the United States of America. 2011;108 Suppl 1:4516-22.

9. Callahan BJ, McMurdie PJ, Rosen MJ, Han AW, Johnson AJ, Holmes SP. DADA2: High-resolution sample inference from Illumina amplicon data. Nat Methods. 2016;13(7):581-3.

10. Caporaso JG, Kuczynski J, Stombaugh J, Bittinger K, Bushman FD, Costello EK, et al. QIIME allows analysis of high-throughput community sequencing data. Nat Methods. 2010;7(5):335-6.

11. Davis NM, Proctor DM, Holmes SP, Relman DA, Callahan BJ. Simple statistical identification and removal of contaminant sequences in marker-gene and metagenomics data. Microbiome. 2018;6(1):226.

12. Lewis ZT, Bokulich NA, Kalanetra KM, Ruiz-Moyano S, Underwood MA, Mills DA. Use of bifidobacterial specific terminal restriction fragment length polymorphisms to complement next generation sequence profiling of infant gut communities. Anaerobe. 2013;19:62-9.

13. Team RC. R: A Language and Environment for Statistical Computing. Vienna, Austria: R Foundation for Statistical Computing; 2023.

14. McMurdie PJ, Holmes S. phyloseq: an R package for reproducible interactive analysis and graphics of microbiome census data. PloS one. 2013;8(4):e61217.

15. Linzer DA, Lewis JB. poLCA: AnRPackage for Polytomous Variable Latent Class Analysis. Journal of Statistical Software. 2011;42(10).

16. Buuren Sv, Groothuis-Oudshoorn K. mice: Multivariate Imputation by Chained Equations inR. Journal of Statistical Software. 2011;45(3).

17. Langfelder P, Horvath S. Eigengene networks for studying the relationships between co-expression modules. BMC Syst Biol. 2007;1:54.

18. Muthén B, Asparouhov T. Causal Effects in Mediation Modeling: An Introduction With Applications to Latent Variables. Structural Equation Modeling: A Multidisciplinary Journal. 2014;22(1):12-23.

19. Peschel S, Muller CL, von Mutius E, Boulesteix AL, Depner M. NetCoMi: network construction and comparison for microbiome data in R. Brief Bioinform. 2021;22(4).

20. Friedman J, Alm EJ. Inferring correlation networks from genomic survey data. PLoS Comput Biol. 2012;8(9):e1002687.
